# Kaempferol prevents aseptic loosening via enhance the Wnt/β-catenin signaling pathway in vitro and in vivo

**DOI:** 10.1186/s40001-023-01469-w

**Published:** 2023-11-09

**Authors:** Wenkui Qiu, Zhenghui Li, Zhenyan Su, Lichao Cao, Lei Li, Xi Chen, Wanhong Zhang, Yanqing Li

**Affiliations:** 1 Department of Orthopedics, Kaifeng Central Hospital, Kaifeng, 475000 Henan People’s Republic of China; 2 Department of Neurosurgery, The Third Affiliated Hospital of Zhengzhou University, Zhengzhou University, Zhengzhou, 450052 Henan People’s Republic of China; 3 School of Life Sciences, Henan University, Kaifeng, 475000 Henan People’s Republic of China; 4 Department of Internal Medicine 3-Rheumatology and Immunology, Friedrich-Alexander Universität Erlangen-Nürnberg (FAU), Universitätsklinikum Erlangen, 91054 Erlangen, Germany; 5 Department of Neurosurgery, Kaifeng Central Hospital, Kaifeng, 475000 Henan People’s Republic of China

## Abstract

Kaempferol has demonstrated notable positive effects on the osteogenic differentiation of mesenchymal stem cells (MSC) and osteoblasts. A substantial body of research has emphasized the role of dislodged titanium particles in aseptic loosening following joint replacement surgery. This study predominantly investigates the suppressive influence of Kaempferol on osteolysis induced by titanium (Ti) alloy particles. In vitro investigations disclosed that Kaempferol effectively enhanced mineralization and alkaline phosphatase (ALP) activity in bone-marrow mesenchymal stem cells exposed to Ti particles. In addition, we conducted a comprehensive analysis of osteogenic differentiation microarray data_sets (GSE37676, GSE79814, and GSE114474) to identify differentially expressed genes. Significantly, Kaempferol upregulated the expression of critical osteogenic markers, including Runt-related transcription factor 2 (Runx2), osteocalcin (OCN), osterix/Sp-7, and β-catenin. In vivo experiments, including H&E staining and Immunohistochemistry, provided compelling evidence that Kaempferol exerted a robust inhibitory effect on periprosthetic osteolysis in mice, with particularly pronounced results at higher doses. Moreover, it elevated the expression levels of osteogenic factors and Wnt/β-catenin signaling components. These findings collectively indicate that Kaempferol mitigates the hindrance to osteogenesis posed by titanium particles by activating the Runx2 and Wnt/β-catenin signaling pathways. This research lays a solid foundation for the prospective utilization of Kaempferol in the management of aseptic loosening following arthroplasty, offering promising therapeutic potential.

**Supplementary Information:**

The online version contains supplementary material available at 10.1186/s40001-023-01469-w.

## Introduction

Artificial joint implantation is considered the best treatment option for osteoarthritis and femoral head necrosis. However, the appearance of aseptic loosening plagues the outcome of surgical treatment [1]. Recent studies have shown that among the various particles shed by implants, titanium (Ti) alloy particles are one of the main causes of the onset and development of joint laxity, while other particles include bone cement, polyethylene, and ceramics [2, 3]. Titanium alloy particles in joint replacement implants may cause inflammation and bone resorption at the interface between the surface of the prosthesis and its adjacent bone, ultimately leading to aseptic loosening of the implant [4]. With the development of biomaterials and prosthesis design, the types of particles produced by artificial arthroplasty have been greatly reduced, but in many patients, titanium particles are still the cause of aseptic loosening after arthroplasty. In addition, as pluripotent stem cells, bone-marrow mesenchymal stem cells (BMSC) can differentiate into osteoblasts, adipocytes, and chondrocytes. In addition, they are widely used in tissue engineering, cell therapy, and gene therapy [5]. Currently, promoting bone formation by inhibiting osteolysis and promoting osteogenic differentiation of BMSC in the presence of titanium particles have become very promising approaches [6].

Kaempferol (3,4',5,7-tetrahydroxyflavone) is a natural flavanol found in a variety of plants and plant foods, such as tea, apples and legumes [7]. Studies have shown that Kaempferol is able to inhibit tumors, anti-inflammatory and many other effects [8, 9]. In addition, kaempferol has been shown to promote osteoblast differentiation and mineralization [10, 11]. Another study also showed that Kaempferol was effective in promoting osteogenesis in ovariectomized rats [12]. Although the role of Kaempferol in inhibiting osteoclastic bone resorption and inducing osteogenic differentiation has been studied, the mechanism of action of Kaempferol on osteogenic differentiation of bone-marrow mesenchymal stem cells in titanium alloy particle-induced osteolysis remains elusive.

In addition, previous studies have demonstrated that the Wnt/β-catenin pathway is critical for the regulation of several cellular activities, such as cell differentiation fate, proliferation, migration, and polarity [13]. The Wnt/β-catenin signaling pathway also plays a key role in the osteogenic differentiation of BMSCs [14, 15]. Jing et al. reported that in osteoporosis, the Wnt/β catenin pathway and osteogenic differentiation of BMSCs were inhibited in osteoporosis [16]. In mechanism, activation of canonical Wnt signaling leads to cytoplasmic stabilization, increased β-catenin translocation to the nucleus, binding to the T-cell factor/lymphocyte enhancer binding factor (Tcf-4/Lef-1) transcription factor, and regulation of downstream target gene expression [17]. In addition, alkaline phosphatase (ALP) is often used as a marker of osteoblast development [18]. During osteogenic differentiation, ALP is considered to be an important master gene controlling osteoblast differentiation and the expression of certain β-catenin targets [19, 20].

A previous study showed that Kaempferol promotes osteogenic differentiation [21]. However, the effects of Kaempferol in vivo and in vitro on aseptic loosening experimental models are not fully understood. Therefore, in the present study, we investigated the effect of Kaempferol on the inhibition of titanium alloy particle-induced osteolysis in vivo and in vitro. The results showed that Kaempferol ameliorated the osteogenesis inhibited by titanium particles by a mechanism that may be related to the activation of the Wnt/β-catenin pathway. These results suggest that Kaempferol may be a useful candidate for the treatment of aseptic loosening after arthroplasty.

## Materials and methods

### Study design

**Fig. 1:**
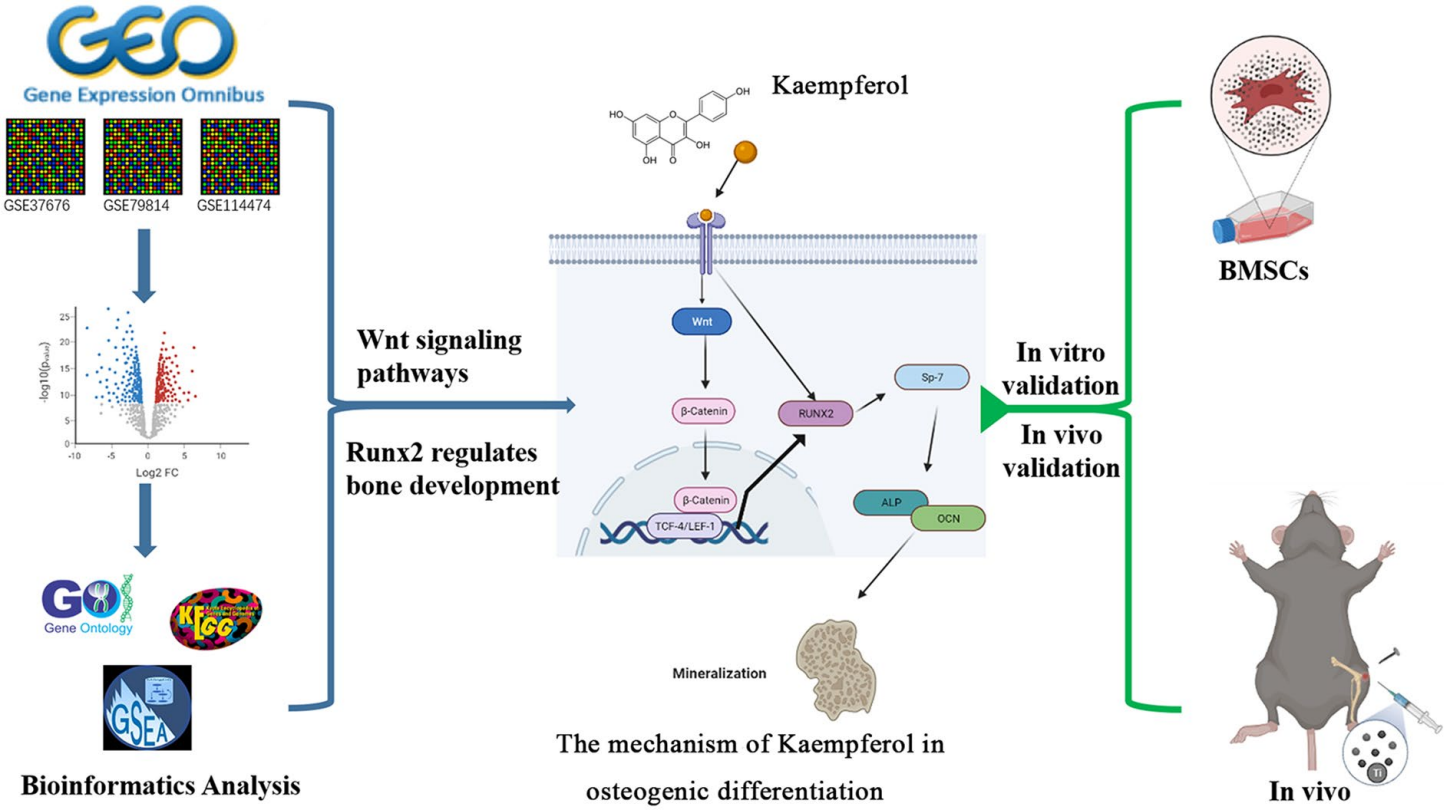
Flow diagram of the study. *GO* Gene Ontology, *KEGG* Kyoto Encyclopedia of Genes and Genomes, *GSEA* gene set enrichment analysis

As shown in Fig. 1, to identify the key pathways and related factors in the process of osteogenic differentiation, we first performed a comprehensive bioinformatics analysis of osteogenic differentiation-related RNA-sequence data. The results showed that Wnt signaling pathways and Runx2 regulates bone development etc. play key roles in this process. Based on these results, in subsequent experiments, we simulated the occurrence of aseptic loosening in vivo and in vitro using titanium alloy particles and Ti-pin models, respectively, and investigated the effects of kaempferol on aseptic loosening and related mechanisms.

### Microarray data information and identification of DEGs

The microarray data set selection criteria are as follows: 1. gene expression data from osteogenic differentiation cultures of mouse bone-marrow mesenchymal stem cells (BMSCs) or osteoblast (MC3T3-E1) were included, excluding osteogenic differentiation-related samples from other species; 2. Each group in the array contains at least 3 samples. respectively; 3. Duration of osteogenic differentiation is at least 14 days; 4. Inclusion of > 5000 genes on the GEO platform. As shown in Table 1, 3 data sets were finally included in this study, and the data sets named GSE37676 [22], GSE79814 [23] and GSE114474 [24] were downloaded from the GEO database (https://www.ncbi.nlm.nih.gov/geo).

**Table 1:** Details of the GEO osteogenic differentiation data

References	Organism	GEO	Platform	Compared groups
Noushin et al. (2012)	Mus	GSE37676	GPL1261	Mc3T3 AA vs control
Meyer et al. [23]	Mus	GSE79814	GPL13112	MSC B15 vs MSC D0
Maria et al. (2018)	Mus	GSE114474	GPL13112	Osteogenic vs Adipogenic

Mc3T3: Osteoblast precursor cell line derived from mouse cranial vault; AA: Ascorbic Acid; B15: treated with osteogenic medium for 15 days

### RNA-seq and functional enrichment analysis

For the comprehensive bioinformatics analysis of the gene data sets, we first normalized the data using the R package "limma" [25], and genes with corrected *P* values < 0.05 and |log fold change (FC)|> 1 were considered differentially expressed genes (DEGs). The RobustRankAggreg (RRA) R package ( https://cran.rstudio.com/bin/windows/contrib/3.5/RobustRankAggreg_1.1.zip) was used to integrate the results of these three data sets, and the integrated list of up- and down-regulated DEGs was saved for subsequent functional analysis. Subsequently, the up- and down-regulated DEGs were used by Metascape (http://metascape.org/) for gene ontology (GO terms) and KEGG (Kyoto Encyclopedia of Genes and Genomes) pathway enrichment analysis to discover enrichment for specific molecular functions, biological processes and terms related to cellular components [26]. In addition, to further investigate the potential mechanism of osteogenic differentiation, we performed Gene Set Enrichment Analysis (GSEA) on the RNA-seq data. The gene set "c2. Cp. Kegg. V7.2. Symbols.gmt" was selected as the reference gene set, false discovery rate (FDR) < 0.25 and *P* value < 0.05 were considered significantly enrichment.

### Preparation of metal particles

Ti-alloy particles were purchased from Zimmer Biomet with an average size of 4.5 µm and a surface area of 0.5 m^2^/mg. To eliminate the possible presence of bacteria and other microorganisms, the particles were sterilized at 180 °C for 45 min, soaked in 95% ethanol for 48 h [27], washed 5 times in sterile phosphate-buffered saline (PBS), and then stored at room temperature. In the in vitro experiments, the Ti-alloy particles were added to the medium. The 0.1% concentration of the particle suspension consisted of ~ 6 × 10^5^ particles, corresponding to approximately 30 µg/ml particles. Therefore, 0.5% included ~ 3 × 10^6^ particles (150 µg/ml) and 1% included ~ 6 × 10^6^ particles (300 µg/ml). In the in vivo experiments, the 10 µl Ti suspension contained 4 × 10^4^ particles of Ti in normal saline, corresponding to approximately 4 × 10^6^ particles (200 µg/ml) [28].

### Cell culture and osteogenic differentiation of BMSCs

BMSCs of C57BL/6 J mice were obtained from Cyagen (Cat. No. MUBMX-01001). Flow cytometric analysis identified that the cells were CD29-, CD44- and Sca-1-positive cells (> 70%), and CD117-negative (< 5%). MSCs were plated in 25-cm^2^ flasks (BD Biosciences) at densities of 1–3 × 10^5^ cells/cm^2^ and cultured with α-minimum essential medium (Invitrogen; Thermo Fisher Scientific, Inc.) supplemented with 10% fetal bovine serum and 1% antibiotics mixture (10,000 U penicillin and streptomycin) in a humidified atmosphere with 5% CO_2_ at 37 °C. The culture medium was renewed every 2 days. A confluent monolayer was obtained after 2 weeks.

For osteogenic differentiation, the cells were transferred to a 12-well plate at a concentration of 7.5 × 10^5^ cells/well, and incubated for 12 h. The medium with 0.1%, 0.5%, and 1% concentration of Ti-alloy particles was added. After 48 h, the medium was changed to MSC osteogenic differentiation medium (Cyagen) with a low concentration (10 μM), or high concentration (30 μM) of Kaempferol (Sigma-Aldrich; St. Louis, MO) [29]. The osteogenic differentiation medium (Cyagen) was composed of 10% fetal bovine serum, 1% penicillin–streptomycin, 1% glutamine, 0.2% ascorbate, 1% β-glycerophosphate and 0.01% dexamethasone. Then, cells were incubated for 21 days and the medium was renewed every 3 days. Osteogenic differentiation was examined by determining alkaline phosphatase (ALP) activity, Alizarin Red S staining, RT-qPCR, and western-blot analysis with specific antibodies [30].

### Experimental animals and treatments

Mice were approved for use by the Animal Protection and Use Committee of Kaifeng Central Hospital Affiliated to Xinxiang Medical university. All the experimental methods were conducted as described previously. [28, 34], totally 30 mice were anesthetized intraperitoneally with Nembutal (0.6% sodium pentobarbital, 60 mg/kg) to expose the tibial plateau. A canal with a diameter of 3 mm was created with a hand drill. Each canal was injected with 10 µl of Ti alloy pellet suspension (4 × 10^4^ pellets of Ti in normal saline) and implanted with a Ti pin. Then, 20 µl Ti particles were injected into the joint capsule every 2 weeks. Furthermore, Somsak et al. showed that kaempferol significantly inhibited malaria at a dose of 10 mg/kg (*P* < 0.05) [31]. Therefore, in our experiments, mice in the control group drank water ad libitum, and mice in the Kaempferol group received oral Kaempferol (5.0 mg/kg/day and 10.0 mg/kg/day) twice daily[28]. After 7 days, the mice were euthanized by 100% CO2 for 5 min, and death was determined after the animals did not move and did not breathe for 2 min. Then, bone formation around the prosthesis was analyzed by histology. The mechanical force of the Ti prosthesis was determined by a pull-out test.

### ALP activity assay

ALP activity of osteoblasts was measured quantitatively using an alkaline phosphatase kit (Sigma-Aldrich; Merck KGaA). Culture supernatants were collected on days 7, 14, and 21, centrifuged at 1000 rpm for 10 min, and 5 µl was transferred to a 96-well plate. Distilled deionized water (5 µl) was added to the blank wells and 5 µl of phenol standard (0.1 mg/ml) was added to the standard wells. Subsequently, 50 µl of buffer solution and 50 µl of substrate solution were added and then incubated for 15 min at 37 °C. After thorough mixing, 150 µl of the color developer was added to each well. The OD520 nm was measured using a spectrophotometer. The ALP activity was determined by comparing the absorbance values.

### Staining and quantification of Alizarin Red S

After 21 days of osteogenic differentiation, the level of BMSC mineralization was assessed by Alizarin Red S staining. First, cells were fixed with 10% (v/v) formalin for 15 min at room temperature, washed twice with PBS and stained with 1 ml Alizarin Red S (pH 4.2; Cyagen) at room temperature for 15 min. Afterward, the stained cells were washed with distilled water to remove the non-specific staining and then incubated with 10% (w/v) cetylpyridinium chloride in 10 mM sodium phosphate (pH 7.0) for 15 min. The samples were then transferred to 96-well plates and the absorbance at 562 nm was measured by spectrophotometer. The concentration of Alizarin Red S in the samples was determined by comparing the absorbance values with those obtained from Alizarin Red S standards.

### Reverse transcription–quantitative PCR (RT–qPCR)

The cells were collected after 21 days of osteogenic differentiation, and total RNA was extracted from the cells according to the instructions of the Omega® E.Z.N.A. total RNA Kit, and the ratio of 260/280 absorbance was calculated to assess RNA purity (NanoDrop; Thermo Fisher Scientific, Inc.). Then, 1 μg of total RNA was used for first-strand cDNA synthesis (Takara, Inc.). A total reaction volume of 15 μl was prepared before amplification, including 1 μl cDNA, 7.5 μl 2X SYBR® Green Real-Time PCR Master Mix (Takara, Japan), 0.5 μM of each primer, and sterile distilled water. The relative expression levels of each indicator gene were calculated by the 2-ΔCt method. Primers for the osteogenic marker genes alkaline phosphatase (ALP), runt-related transcription factor 2 (Runx2), osteocalcin (OCN), Wnt/β-catenin signaling pathway (β-catenin, Lef-1 and Tcf-4) and reference gene GAPDH are shown in Table 2.

**Table 2:** Primer sequences for ALP, β-catenin, Lef-1, Tcf-4 and GAPDH

Gene		Sequences ( 5′–3′)	Product size (bp)
Runx2	Forward	CGGGTCTCCTTCCAGGAT	18
	Reverse	GGGAACTGCTGTGGCTTC	18
OCN	Forward	GAGGGCAATAAGGTAGTGAA	20
	Reverse	CATAGATGCGTTTGTAGGC	19
ALP	Forward	CCCAAAGGCTTCTTCTTGC	19
	Reverse	GCCTGGTAGTTGTTGTGAG	19
β-catenin	Forward	ACAGGGTGCTATTCCACGAC	20
	Reverse	CTGCACAAACAATGGAATGG	20
Lef-1	Forward	GCCACCGATGAGATGATCCC	20
	Reverse	TTGATGTCGGCTAAGTCGCC	20
Tcf-4	Forward	ATGGCCCAAGTAGTGATGTCT	21
	Reverse	CAAACACGTCGGTCTCATACA	21
GAPDH	Forward	CAATGACCCCTTCATTGACC	20
	Reverse	TGGACTCCACGACGTACTCA	20

*GAPDH* glyceraldehyde phosphate dehydrogenase, *ALP* alkaline phosphatase, *Lef-1* lymphoid enhancer binding factor 1, *OCN* osteocalcin, *Runx2* runt-related transcription factor 2, *Tcf-4* transcription factor 4

### Western blot analysis

For in vitro studies, cells were washed with cold PBS after 21 days of osteogenic differentiation. For in vivo studies, the soft tissue around the implants was ground in liquid nitrogen. Proteins were then extracted with RIPA buffer containing 1 mM phenylmethylsulfonyl fluoride. After centrifugation, protein concentration was measured with bicinchoninic acid, and 40 μg of total protein was separated by sodium dodecyl sulfate–polyacrylamide gel electrophoresis and transferred to polyvinylidene fluoride membranes. The membranes were blocked in 5% dry skim milk for 2 h and then incubated with primary antibodies (Runx2, ab92336, 1:500; OCN, ab76690, 1:1000; Sp-7, ab209484, 1:1000; β-catenin, ab6302, 1:1000) and the β-actin (1:20,000) overnight at 4 °C. After incubation with secondary antibodies (1:2,000), the membranes were exposed to enhanced chemiluminescence ECL reagents and analyzed using a detection system (PerkinElmer, Inc.). The protein bands were analyzed semi-quantitatively using ImageJ software (LI-COR Biosciences).

### Histological staining

Tissue samples were fixed in paraformaldehyde (4%) at 4 °C for 24 h and then decalcified with EDTA (4%) for 30 days. After decalcification, samples were dehydrated in a series of graded ethanol’s and embedded in paraffin at 60˚C. Sections of 5 µm thickness were cut using the RM2235 rotary minicomputer instrument (Leica Microsystems, Inc.) centered on the long axis of the tibia. The sections were then stained with hematoxylin at 23˚C for 3 min and 0.5% water-soluble eosin at 23 °C for 5 min (H&E). For immunohistochemical staining, sections were incubated overnight at 4˚C using Runx2, OCN, and β-catenin, and blocked with goat serum (Goat Anti-Rabbit IgG Polymer, Mesozoic, Beijing, China) for 40 min at 23 °C. Sections were analysed by microscope (DM2000 LED; Leica Microsystems, Inc.)

### Ti prosthesis steadiness measured by a pullout test

Following euthanasia, the bone formation around the implant was measured by the mechanical force required to pull out the Ti-pin. First, the soft tissues around the tibia were carefully removed and the head of the Ti implant was exposed. Each tibia was fixed to a special clamp with dental cement, which was designed to align the long axis of the implants with the long axis of the HP-100 control electronic universal testing machine (Yueqing Zhejiang Instrument Scientific Co., Ltd.). After the tibia and the custom fixture were properly positioned, the HP-100 device pulled the Ti implant out of the tibia at a rate of 2 mm/min. The load values were measured automatically using appropriate software (Edburg; Yueqing ALIYIQI Instrument Co., Ltd.).

### Statistical analysis

Results are expressed as the means ± standard deviation of at least three independent experiments. Two groups were compared using the Tukey’s *t* test. Comparisons of multiple groups were performed by one-way analysis of variance and Dunnett’s T3 test (without assuming equal variance). *P* < 0.05 was considered to indicate a statistically significant difference. SPSS 19.0 (IBM Corp.) was used for statistical analysis. Graphs were prepared using GraphPad Prism (version 6.0 for Windows).

## Results

### Identification of DEGs in osteogenic differentiation

**Fig. 2:**
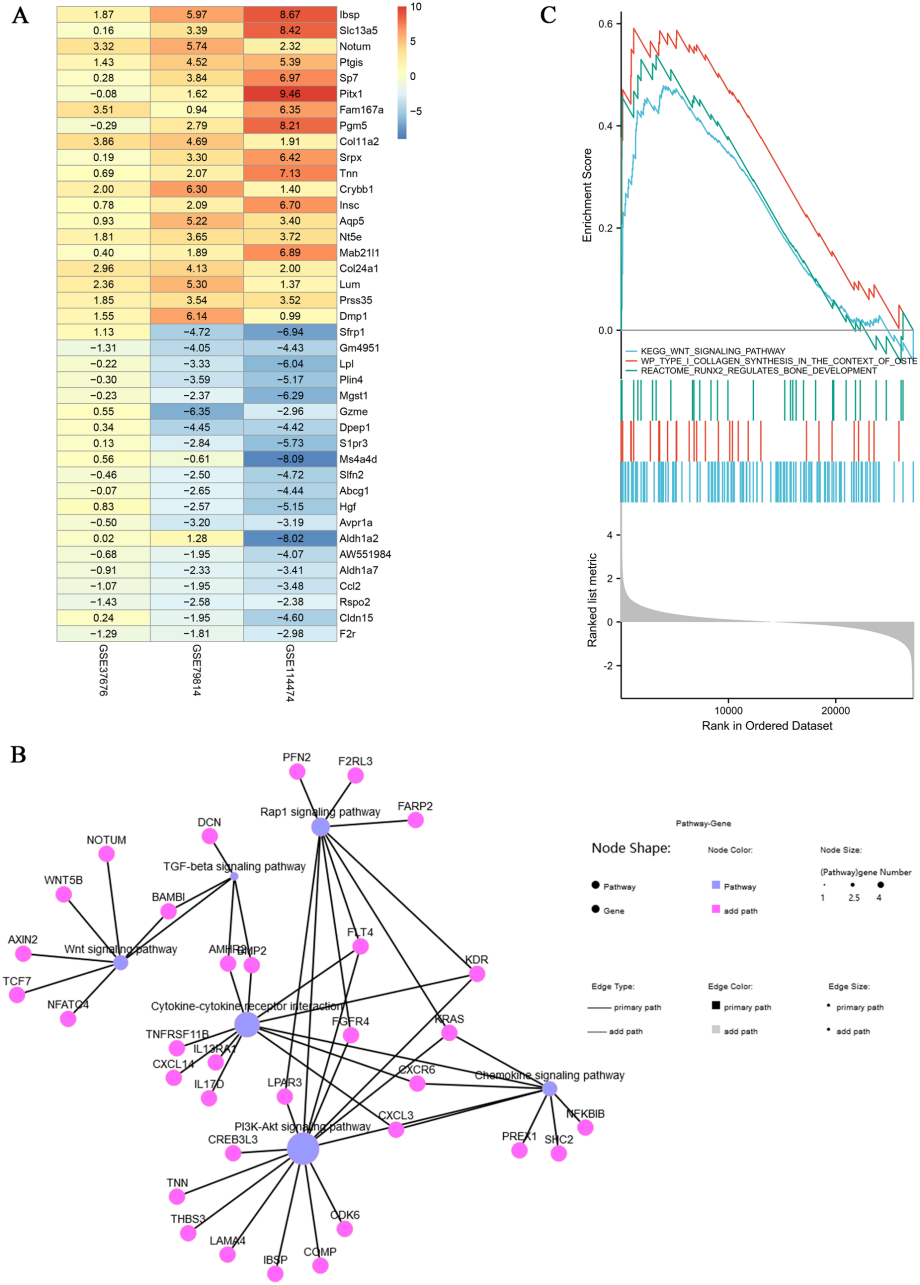
Comprehensive bioinformatics analysis of osteogenic differentiation-related data sets in GEO. **A** Log FC heatmap of each expression microarray. The abscissa represents the GEO IDs, the ordinate represents the gene name, the red represents log FC > 0, the blue represents log FC < 0 and the value in the box represents the log FC value. **B** Network map of KEGG enrichment analysis (Blue dots are upregulated signaling pathways, purple dots are pathway-related genes). **C** Results of GSEA related to bone formation

The osteogenic differentiation microarray data sets named GSE37676, GSE79814 and GSE114474 were first normalized (Additional file 1: Fig. S1A, B). Then, DEGs were identified by the limma R package with *P* < 0.05, |log fold change (FC)|> 1. As shown in Additional file 1: Fig. S1C, GSE37676 contained 695 DEGs, containing 390 up-regulated and 305 down-regulated genes. GSE79814 contained 2432 DEGs containing 1299 up-regulated genes and 1133 down-regulated genes (Additional file 1: Fig. S1E). GSE114474 contained 933 DEGs containing 362 up-regulated genes and 571 down-regulated genes (Additional file 1: Fig. S1G). In addition, the heatmap of DEGs and their top 100 genes for each of the three data sets are shown in Additional file 1: Fig. S1D, F and H. In total, 11,117 genes were selected to further analysis (Additional file 2: Fig. S2A). After that, the RRA package was used to screen the DEGs of the three data sets with P < 0.05, |log fold change (FC)|> 1. By rank analysis, a total of 301 up-regulated genes and 224 down-regulated genes were obtained, and the clustering heat map of the top 40 genes is shown in Fig. 2A.

### Functional enrichment analysis

The Metascape database annotation tools performed GO function annotation and KEGG pathway analysis of the integrated DEGs. As shown in Additional file 2: Fig. S2B, C, the GO function annotation, including three subontologies: BP, CC, and MF. Respectively, in BP, upregulated genes were mainly enriched in extracellular structure organization, tissue morphogenesis and ossification, downregulated genes were mainly enriched in response to bacterium, blood vessel development and vasculature development. In CC, upregulated genes were mainly enriched in extracellular matrix, collagen-containing extracellular matrix and apical part of cell, downregulated genes were mainly enriched in extracellular matrix, collagen-containing extracellular matrix and vacuole. In MF, upregulated genes were mainly enriched in structural molecule activity, extracellular matrix structural constituent and calcium ion binding, downregulated genes were mainly enriched in receptor ligand activity, receptor regulator activity, and oxidoreductase activity. Furthermore, the results of KEGG showed that the integrated DEGs were mainly enriched in cytokine–cytokine receptor interaction, PI3K–Akt signaling pathway, calcium signaling pathway, chemokine signaling pathway, Wnt signaling pathway, and PPAR signaling pathway (Table 3). In particular, Wnt signaling pathway and cytokine–cytokine receptor interaction play an important role in this (Fig. 2B, Additional file 2: Fig. S2D). Similarly, the GSEA analysis also showed that the osteogenic differentiation was closely associated with “Wnt signaling pathways”, “type I collagen synthesis in the context of osteogenesis imperfect” and “Runx2 regulates bone development” (Fig. 1C). Therefore, in our subsequent experiments, we verified the effect of kaempferol on Wnt signaling pathways and Runx2 regulates bone development in vitro and in vivo.

**Table 3:** Kyoto Encyclopedia of Genes and Genomes (KEGG) pathway analysis of integrated DEGs

Pathway	ID	Gene count	Log *P*-value	Genes
Up-regulated KEGG				
Wnt signaling pathway	hsa04310	7	− 2.82093	NFATC4,ROR2,TCF7,AXIN2, BAMBI,WNT5B,NOTUM
Protein digestion and absorption	hsa04974	10	− 7.48275	COL3A1,COL11A1,COL11A2,COL13A1,SLC8A2, XPNPEP2,KCNK5,SLC7A7,COL22A1,COL24A1
Nitrogen metabolism	hsa00910	3	− 3.15626	CA6,CA9,CA12
Rap1 signaling pathway	hsa04015	8	− 2.69188	FGFR4,FLT4,KDR,KRAS,PFN2, F2RL3,FARP2,LPAR3
PI3K-Akt signaling pathway	hsa04151	12	− 3.35898	CDK6,COMP,FGFR4,FLT4,IBSP,KDR, KRAS,LAMA4,THBS3,LPAR3,TNN,CREB3L3
Human papillomavirus infection	hsa05165	11	− 3.08673	CDK6,COMP,IBSP,KRAS,LAMA4,TCF7, THBS3,AXIN2,TNN,WNT5B,CREB3L3
Focal adhesion	hsa04510	8	− 2.85175	COMP,FLT4,IBSP,KDR,LAMA4, THBS3,SHC2,TNN
ECM-receptor interaction	hsa04512	5	− 2.78273	COMP,IBSP,LAMA4,THBS3,TNN
Pathways in cancer	hsa05200	15	− 3.17301	BDKRB1,BDKRB2,BMP2,CDK6,FGFR4,FLT4, IL13RA1,KRAS,LAMA4,PTCH1,TCF7,AXIN2, F2RL3,LPAR3,WNT5B
Basal cell carcinoma	hsa05217	5	− 3.15797	BMP2,PTCH1,TCF7,AXIN2,WNT5B
Breast cancer	hsa05224	7	− 2.92668	CDK6,FLT4,KRAS,TCF7,AXIN2, SHC2,WNT5B
Cushing syndrome	hsa04934	6	− 2.14083	CDK6,PDE8A,TCF7,AXIN2, WNT5B,CREB3L3
Hepatocellular carcinoma	hsa05225	6	− 2.01463	CDK6,KRAS,TCF7,AXIN2, SHC2,WNT5B
Cytokine-cytokine receptor interaction	hsa04060	10	− 2.775	AMHR2,BMP2,FLT4,CXCL3,IL13RA1,KDR, TNFRSF11B,CXCL14,CXCR6,IL17D
TGF-beta signaling pathway	hsa04350	5	− 2.6331	AMHR2,BMP2,DCN,FMOD,BAMBI
Chemokine signaling pathway	hsa04062	7	− 2.38828	CXCL3,KRAS,NFKBIB,CXCL14, CXCR6,SHC2,PREX1
Biosynthesis of amino acids	hsa01230	4	− 2.18385	CTH,ENO2,PGAM2,PYCR1
cGMP-PKG signaling pathway	hsa04022	6	− 2.11711	ATP2A1,BDKRB2,KCNMA1,NFATC4, SLC8A2,CREB3L3
Down-regulated KEGG				
Neuroactive ligand-receptor interaction	hsa04080	10	− 4.7048802	ADCYAP1R1,ADM,AGT,AVPR1A,C3AR1, S1PR3,F2R,GABRA4,PRL,CALCRL
Pathways in cancer	hsa05200	10	− 3.1714531	AGT,CEBPA,COL4A1,COL4A2,F2R,HGF, IL5,MGST1,MGST3,CXCL12
Cytokine-cytokine receptor interaction	hsa04060	8	− 3.5862507	HGF,IL5,PRL,CCL2,CCL7,CCL15,CXCL11, CXCL12
PI3K-Akt signaling pathway	hsa04151	8	− 3.1640003	ANGPT1,COL4A1,COL4A2,ERBB4,F2R, HGF,PRL,SPP1
AGE-RAGE signaling pathway in diabetic complications	hsa04933	6	− 4.7698524	AGT,COL4A1,COL4A2,F3, SERPINE1,CCL2
Chemokine signaling pathway	hsa04062	5	-2.4473282	CCL2,CCL7,CCL15,CXCL11, CXCL12
NOD-like receptor signaling pathway	hsa04621	5	− 2.6008533	CASP1,DEFA1,IFI16, MEFV,CCL2
Vascular smooth muscle contraction	hsa04270	5	− 3.1886762	ADM,AGT,AVPR1A, CALCRL,KCNMB4
Phospholipase D signaling pathway	hsa04072	4	− 2.023575	AGT,AVPR1A,F2R,PIP5K1B
Complement and coagulation cascades	hsa04610	4	− 2.9382136	C3AR1,F2R,F3,SERPINE1
Chemical carcinogenesis	hsa05204	4	− 2.9741296	CYP1B1,MGST1,MGST3, SULT1A1
PPAR signaling pathway	hsa03320	4	− 3.0297181	LPL,ADIPOQ,NR1H3,PLIN4
ECM-receptor interaction	hsa04512	3	− 2.030843	COL4A1,COL4A2,SPP1
Ovarian steroidogenesis	hsa04913	3	− 2.5731323	CYP1B1,CYP2J2,AKR1C3
Arachidonic acid metabolism	hsa00590	3	− 2.4057499	CYP2J2,EPHX2,AKR1C3
Metabolism of xenobiotics by cytochrome P450	hsa00980	3	− 2.1782063	CYP1B1,MGST1,MGST3

### Ti-alloy particles inhibit the osteogenesis of BMSCs in vitro

**Fig. 3:**
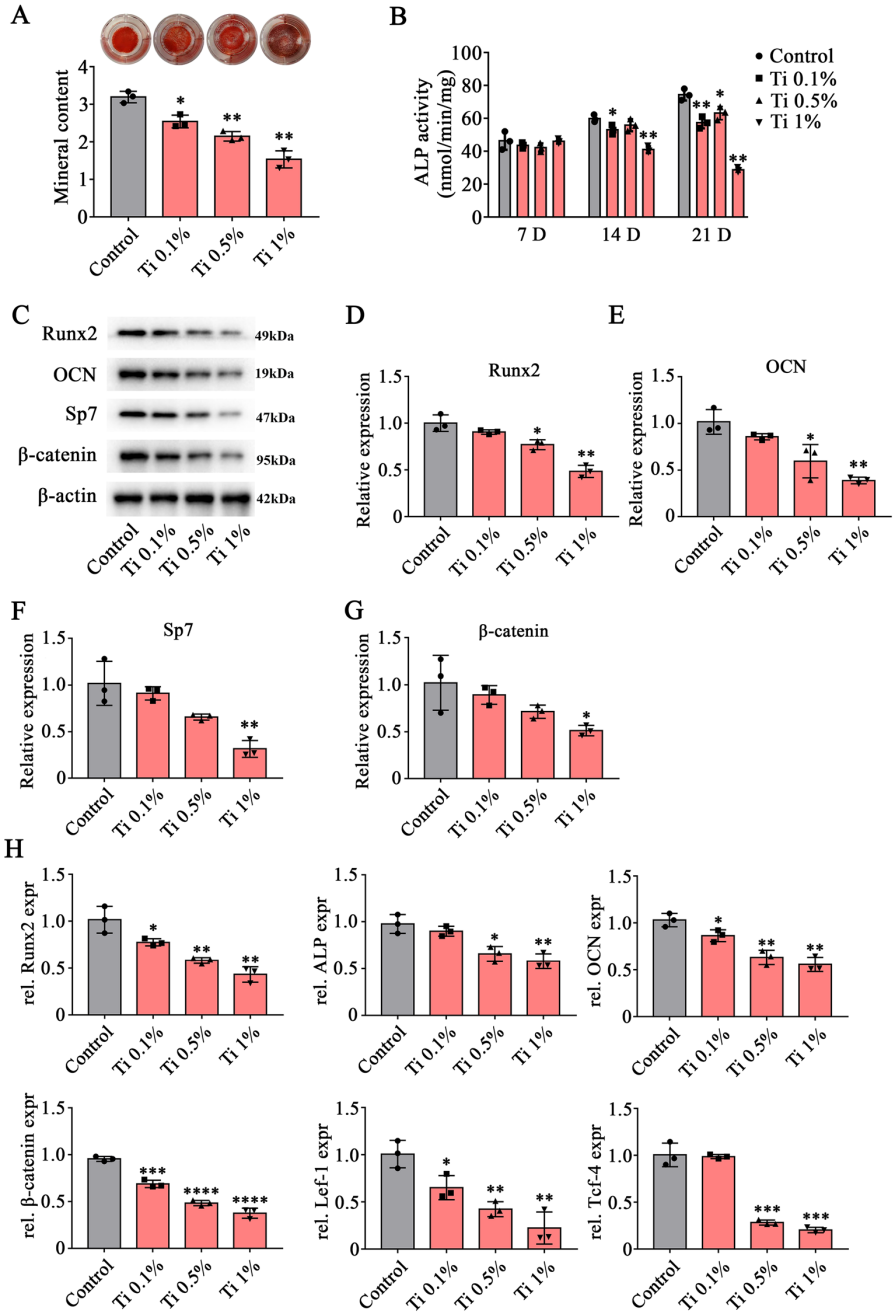
Ti-alloy particles inhibit the osteogenic differentiation of BMSCs in vitro. **A** Alizarin Red S staining and quantitative analysis of calcified matrix in cells cultured with the 0.1%, 0.5% and 1% concentrations of Ti-alloy particles. **B** Expression of ALP in culture medium treated with the 0.1%, 0.5% and 1% concentrations of Ti-alloy particles at 7, 14, and 21 days of osteogenic differentiation. **C**–**G** Expression of Runx2, OCN, Sp-7 and β-catenin were detected by western-blot analysis following treatment with the 0.1%, 0.5% and 1% concentrations of Ti-alloy particles after 21 days of osteogenic differentiation. **H** BMSCs were treated with 0.1%, 0.5% and 1% concentrations of Ti-alloy particles for 24 h. Cell viability was detected by CCK-8 assay. **I** RT-PCR of Runx2, ALP, OCN and β-catenin, Lef-1, Tcf-4 after 21 days of osteogenic differentiation. Data are shown as the mean ± SD. Compared with Control group. *P < 0.05, **P < 0.01, ***P < 0.001 and ****P < 0.0001, *n* = 3

To determine the effects of various concentrations of Ti-alloy particles on BMSCs in vitro, the effects of Ti-alloy particles on osteogenesis and the Wnt/β-catenin signaling pathway were investigated. The results of Alizarin Red S staining revealed that osteogenic differentiation was significantly inhibited after treated with Ti-alloy particles, particularly at the 0.5% and 1% concentrations (Fig. 3A). Following the addition of various concentrations of Ti-alloy particles, ALP activity in the osteogenic differentiation medium was significantly decreased at 21 days, but not at 7 days (Fig. 3B). Notably, at 21 days of osteogenic differentiation, the expression level of osteogenic factors, such as Runx2, OCN, and Sp7 were significantly decreased in the 0.5% and 1% Ti-alloy particles (Fig. 3C–F). In addition, the β-catenin was also inhibited, especially in the 1% concentration (Fig. 3C, G). In addition, the results of RT–PCR also showed that Runx2, ALP, and OCN were significantly decreased. In addition, the level of β-catenin, Lef-1, and Tcf-4, the key factors of the Wnt/β-catenin signaling pathway, were also reduced after treated with Ti-alloy particles (Fig. 3H).

### Kaempferol enhances the osteogenic differentiation of BMSCs cultured with Ti-alloy particles

**Fig. 4:**
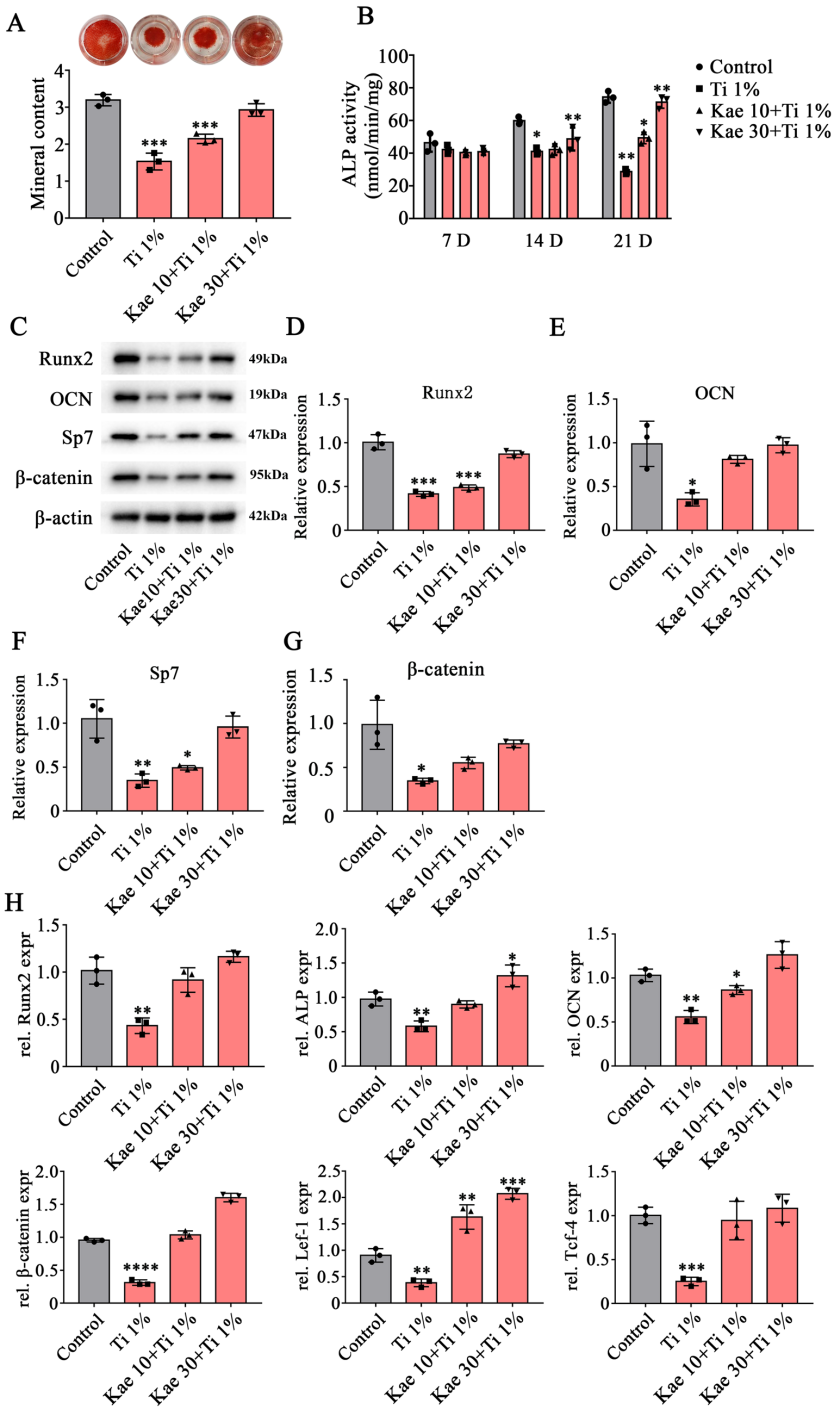
Effects of Kaempferol on osteogenesis in the presence of 1% Ti-alloy particles after 21 days of osteogenic differentiation. **A** Calcified matrix and quantification of Alizarin Red staining intensity of cells treated with Kaempferol and Ti-alloy particles following osteogenic differentiation for 21 days. **B** ALP activity in culture medium treated with Kaempferol and Ti alloy-particles at 7, 14, and 21 days of osteogenic differentiation. **C**–**G** Expression of Runx2, OCN, Sp-7 and β-catenin determined by western-blot analysis following treatment with the Kaempferol after 21 days of osteogenic differentiation. **H** BMSCs were treated with Ti-alloy particles and Kaempferol for 24 h. Cell viability was detected by CCK-8 assay. **I** RT-PCR of Runx2, ALP, OCN and β-catenin, Lef-1, Tcf-4 after 21 days of osteogenic differentiation. Data are shown as the mean ± SD. Compared with Control group. *P < 0.05, **P < 0.01, ***P < 0.001 and ****P < 0.0001, *n* = 3

To verify the effects of Kaempferol on the osteogenesis of BMSCs treated with various concentrations of Ti-alloy particles, Alizarin Red S staining and osteogenic factors was detected after 21 days of osteogenic differentiation. The Alizarin Red S staining revealed that 10 μM Kaempferol cannot reverse the osteolysis caused by Ti-particles. However, it significantly inhibited the occurrence of osteolysis at 30 μM Kaempferol (Fig. 4A). In addition, ALP activity was assayed at 7, 14 and 21 days of osteogenic differentiation. As shown in Fig. 2B, quantitative analysis of ALP activity showed a significant decrease in ALP activity at 14 and 21 days of osteogenic differentiation after treatment with Ti alloy particles, while only the 30 μM Kaempferol showed no significant decrease in ALP activity compared with the control group.

Next, 1% concentration of Ti-alloy particles were used to simulate the aseptic loosening in vitro. We examined osteogenic differentiation and Wnt/β-catenin signaling pathway-related factors after 21 days of osteogenic differentiation (Fig. 4C–G). The results of western-blot showed that the expression of Runx2, OCN, SP7, and β-catenin were suppressed by Ti-alloy particles. In addition, compared with the control group, the expression of Runx2 and SP7 was significantly inhibited after treated with 10 μM Kaempferol. However, in the group of 30 μM Kaempferol, the expression of osteogenic factors Runx2, OCN and Sp7 did not show a significant decrease (Fig. 4C–F). Moreover, the expression of β-catenin did not show a significant decrease (Fig. 4C, G). The results of RT–PCR also showed that the expression of Runx2, ALP and OCN were significantly inhibited by Ti alloy particles. In addition, this inhibition was then reversed after treated with 10 μM and 30 μM Kaempferol. It is worth noting that after Kaempferol treatment, the expression of β-catenin, Lef-1 and Tcf-4 increased significantly (Fig. 4H). These results suggest that Kaempferol can inhibit Ti-alloy particles induced osteolysis by upregulating the Wnt/β-catenin signaling pathway.

### Kaempferol enhances the bone microstructure around the prosthesis

**Fig. 5:**
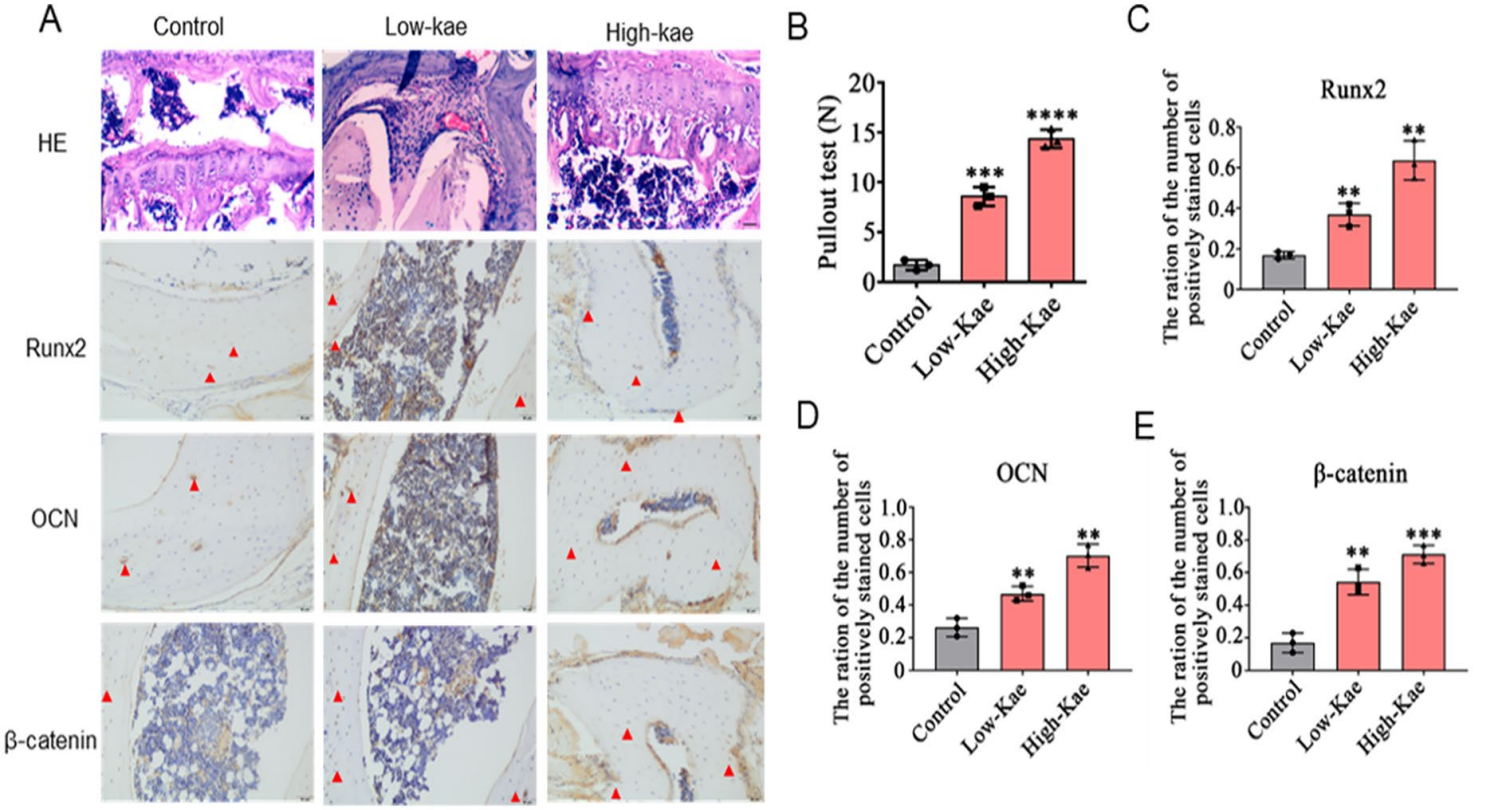
Kaempferol enhances the micro bone formation around the prosthesis in vivo. **A** Histological assessment of periprosthetic tissue (scale bar, 50 μm). **B** Pulling force required to remove the titanium pin implant in tibia with or without Kaempferol. **C**–**E** Histology analysis and semi‑quantitative analysis of the Runx2, OCN, and β-catenin, the blue triangles are positive staining (scale bar, 100 μm). Data are shown as the mean ± SD. Compared with Control group. *P < 0.05, **P < 0.01, ***P < 0.001 and ****P < 0.0001, *n* = 3

To investigate the effects of Kaempferol on bone formation in vivo, H&E and Immunohistochemistry were used to analyze the differences in bone formation among the control, low-dose and high-dose Kaempferol group (Fig. 5). The results of H&E staining indicated that, in the Kaempferol group, the areas of bone resorption were decreased, particularly in the high-dose Kaempferol group (10 mg/kg/day, Fig. 5A). To study the effect of bone formation around the Ti implant on the mechanical force of the prosthesis, the pulling force was detected. The results revealed that the average pulling load of the control group was 1.31 ± 0.58 N. Following treatment with Kaempferol, the pulling load was significantly enhanced in the low-dose Kaempferol group (8.75 ± 0.62N, *P* < 0.01) and the high-dose Kaempferol group (14.28 ± 0.38N, *P* < 0.01). In addition, a significant difference in pulling load was detected between the two Kaempferol groups (Fig. 5B). In mechanism, IHC was used to detect the expression of osteogenic factors, such as Runx2, OCN and β-catenin. Compared with the control group, the expression level of Runx2 and OCN was significantly increased in the Kaempferol group. Similarly, the expression levels of β-catenin were also significantly increased after treated with Kaempferol, especially at high doses (Fig. 5A, C and D).

## Discussion

Currently, joint arthroplasty stands as an established and dependable treatment for osteoarthritis, exhibiting the capability to effectively manage symptoms and reinstate joint functionality [32–34]. Nevertheless, aseptic loosening is the primary factor influencing the success rate of joint replacement. While prior research has highlighted the pivotal role of an osteogenesis–osteolysis imbalance in the vicinity of the prosthesis in aseptic loosening [35, 36], it remains a long-term complication of joint arthroplasty with unclear osteolysis pathogenesis.

Multipotent stem cells, particularly bone-marrow-derived mesenchymal stem cells (BMSCs), have found extensive applications in tissue engineering, cell therapy, and gene therapy due to their superior osteogenic attributes compared to preosteoclasts [5]. In this investigation, we scrutinized the impact of Kaempferol on Ti-alloy particle-induced osteolysis and the associated mechanisms. In addition, an in vivo mouse Ti pin model was established to mimic aseptic loosening and explore the regulatory influence of Kaempferol on periprosthetic bone formation. Following the methodology of previous studies [37], we assessed the expression of osteogenic markers, such as ALP, Runx2, OCN, and Sp-7 to gauge osteogenesis. Moreover, the canonical/non-canonical Wnt signaling pathway, which plays a pivotal role in cell proliferation, differentiation, adhesion, and migration in various mammalian organs and tissues [38], was evaluated for its significance in bone development, homeostasis, osteogenic differentiation, and bone mineralization [39].

Our findings unveiled that Ti alloy particles significantly suppressed ALP activity in the medium and hindered the osteogenic differentiation of BMSCs, particularly at concentrations of 0.5% and 1%. These outcomes align with earlier observations, where higher concentrations of Ti alloy particles correlated with more pronounced inhibition of osteogenesis [3, 40, 41]. Furthermore, key osteogenic differentiation markers, namely, ALP, Runx2, OCN, and Sp-7, exhibited significant reduction post-exposure to Ti alloy particles. Concurrently, the expression levels of factors associated with the Wnt/β-catenin signaling pathway, including β-catenin, Lef-1, and Tcf-4, experienced substantial downregulation. Based on these results, we posit that Ti particles downregulate osteogenic-related factors and impede the Wnt/β-catenin signaling pathway, shedding light on potential mechanisms underlying aseptic loosening in joint arthroplasty. In a series of subsequent experiments, we investigated the impact of Kaempferol on bone-marrow-derived mesenchymal stem cells (BMSCs). Our findings reveal that following 21 days of osteogenic differentiation, a lower concentration (10 μM) of Kaempferol did not effectively counteract the inhibitory effects of Ti alloy particles on osteogenic differentiation. However, a higher concentration (30 μM) of Kaempferol significantly promoted osteogenic differentiation and ameliorated the negative effects of Ti-alloy particles. Notably, this effect was more pronounced at the 30 μM dose. This observation was consistent with Alizarin Red S staining results. Expression levels of key osteogenic differentiation factors and components of the Wnt/β-catenin signaling pathway, including Runx2, OCN, Sp-7, β-catenin, Lef-1, and Tcf-4, were notably upregulated upon treatment with 10 μM and 30 μM Kaempferol, with the 30 μM dose exhibiting the most significant enhancement. Our findings resonate with prior studies, where Kaempferol demonstrated the ability to stimulate osteogenic differentiation of mesenchymal stem cells (MSCs) and osteoblasts while inhibiting osteoclast differentiation, thereby augmenting bone formation capacity [42–45]. Furthermore, Kaempferol exhibited dose-dependent mitigation of dexamethasone-induced inhibition of osteogenic differentiation in MC3T3-E1 osteoblasts, involving the regulation of Runx2 and Osterix expression [46].To further investigate the effect of Kaempferol on aseptic loosening after joint prosthesis replacement, we simulated the occurrence of aseptic loosening by Ti-pin and Ti alloy particles in C57BL/6 J mice. Not completely similar to the results of the in vitro experiments, HE staining showed that high and low doses of Kaempferol significantly increased periprosthetic bone formation. In addition, the results of Tb. Th and Tb. N showed that significant differences were observed only between the high-dose Kaempferol treatment group and the control group. Moreover, in terms of mechanical stress, Kaempferol significantly increased the extraction mechanical force of Ti-pin, which indicated that Kaempferol could effectively resist the occurrence of aseptic loosening after joint replacement caused by Ti alloy particles. Mechanistically, like the in vitro results, both high and low doses of Kaempferol significantly upregulated the expression levels of Runx2, OCN, SP-7, and β-catenin, especially the high dose of Kaempferol, but the results were similar to those of μCT. Thus, it is reasonable to assume that, Ti-alloy particles downregulate the expression levels of osteogenic factors and Wnt/β-catenin signaling pathways, ultimately leading to the inhibition of bone formation. In particular, at high concentrations of Ti particles (possibly exceeding 0.5%), significantly increased periprosthetic osteolysis ultimately leads to the development of aseptic loosening.

The Wnt/β-catenin signaling pathway has been shown to play an important role in the osteogenic differentiation of stem cells [47]. Studies have shown that this pathway is not only involved in the homeostatic control of bone mass, but also plays an important role in aseptic loosening. Beloti et al. found that titanium particles were able to regulate osteogenic differentiation through Wnt/β-catenin signaling of receptor [48]. In addition, Xu et al. found that in titanium particle-induced osteolysis, cranial surface osteolysis was accompanied by an increase in sclerostin levels. In addition, in vitro, osteolysis was also accompanied by increased expression of sclerostin in osteoblasts [49]. However, the detailed mechanism by which Kaempferol prevents aseptic loosening in vivo has not been studied. Previous studies have shown that mesenchymal stem cells and osteoblasts play an important role in regulating bone regeneration [50, 51]. Kaempferol can direct BMSCs toward osteoblast lineages by upregulating the Wnt pathway [30]. However, there are no relevant studies demonstrating the effect of Kaempferol on aseptic loosening. Our findings suggest that titanium particles attenuate the Wnt/β-catenin signaling pathway thereby inhibiting osteogenic differentiation, whereas Kaempferol effectively increases this signaling pathway and increases the osteogenic differentiation capacity of BMSCs. In mechanism, with the upregulation of Wnt/β-catenin signaling pathway, osteogenic-related factors Runx2, OCN and Sp-7 were also upregulated.

In conclusion, Kaempferol effectively inhibited wear particle-associated osteolysis in vivo, and promoted the osteogenic differentiation of BMSCs cultured with Ti-alloy particles in vitro, particularly at the high dose. In addition, the activation of the Wnt/β-catenin signaling pathway may contribute to the osteogenic differentiation and the enhanced expression of Runx2 and bone formation. The further understanding of the molecular mechanisms of Kaempferol will potentially provide a possible application for aseptic loosening in the future.

### Supplementary Information

**Additional file 1: Figure S1.** Normalization and differential analysis of gene expression. **A** Blue represents data before normalization. **B** Red represents data after normalization. **C**, **D** and **E** Volcano plot of DEGs among GSE37676, GSE79814 and GSE114474. F, **G** and **H** Heatmap of the top 40 differentially expressed mRNAs from the GEO microarray GSE37676, GSE79814 and GSE114474

**Additional file 2: Figure S2.** Functional enrichment analysis of osteogenic All reference citation need to be check manually, abbreviation tagged in normal para tag differentiation-related data sets. **A** Venn diagram of mRNA in GSE37676, GSE79814 and GSE114474. **B** Bar plot of GO terms for up/down regulation. GO term name was assigned to *y*-axis and Gene number was assigned to *x*-axis. Biological processes (BP), cellular components (CC) and molecular functions (MF) are distinguished by different colors. **C** GO Chord plot of the relationship between the selected GO terms and their corresponding genes. The left half of the GO chord shows the expression ploidy of the gene (Red is the up-regulated, blue is the down-regulated). The right half represented different GO terms with different colors. **D** Results of KEGG pathway enrichment analyses for up/down-regulated DEGs. Rich factor = count/pop hits. *KEGG* Kyoto Encyclopedia of Genes and Genomes, *DEGs* differently expressed genes, *GO* gene ontology

## Data Availability

These data were derived from the following resource available in public domain: GSE37676 [23], GSE79814 [24] and GSE114474 [25]. In addition, the experiment data that support the findings of this study are available from the corresponding author upon reasonable request.
