# Supplementary figures and images for: Kaempferol prevents aseptic loosening via enhance the Wnt/β-catenin signaling pathway in vitro and in vivo
Source: Eur J Med Res. 2023 Nov 9;28:505. doi: 10.1186/s40001-023-01469-w (PMC10634165; doi:10.1186/s40001-023-01469-w)

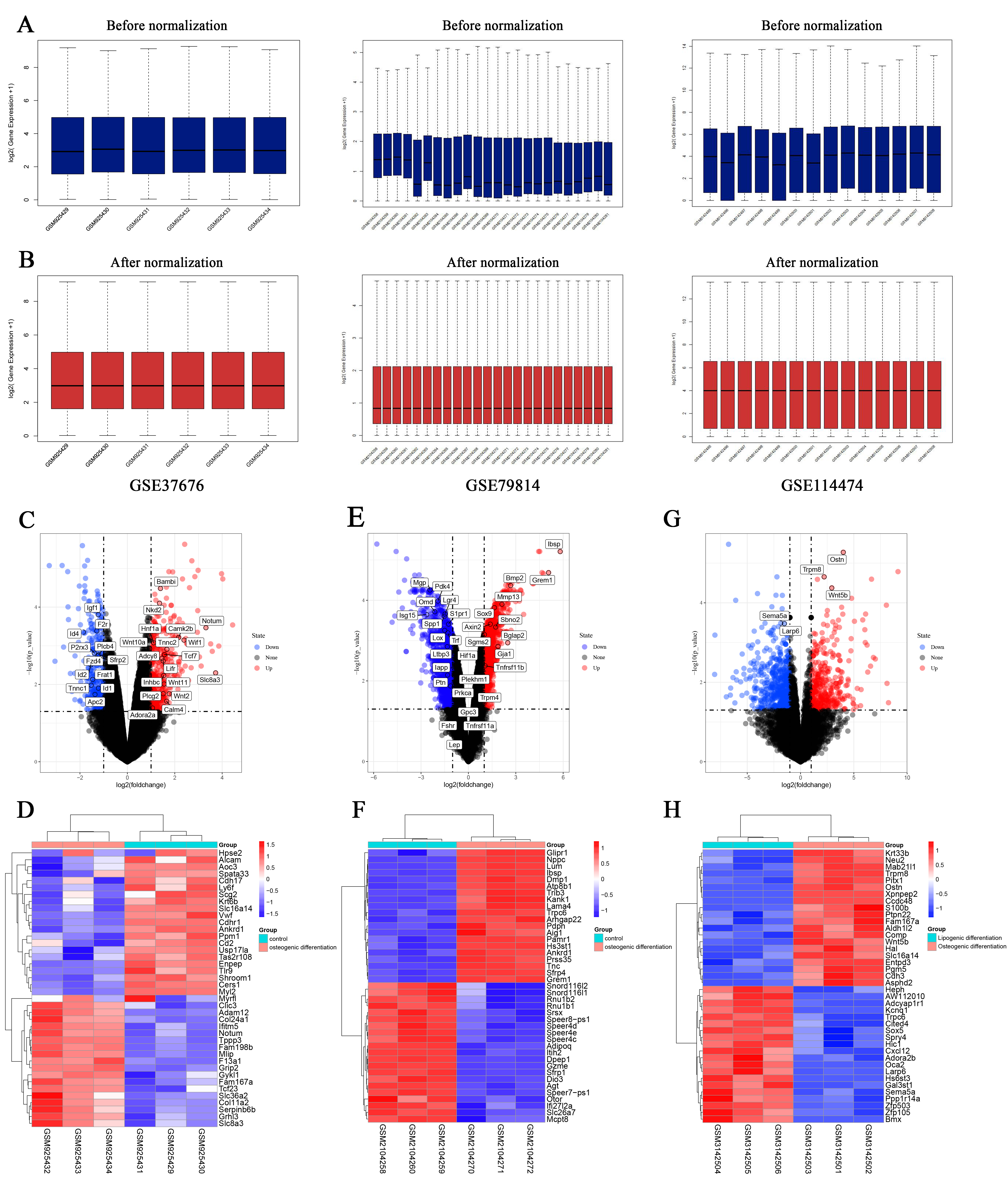

Supplement: Supplementary file 1 — Additional file 1: Figure S1. Normalization and differential analysis of gene expression. A Blue represents data before normalization. B Red represents data after normalization. C, D and E Volcano plot of DEGs among GSE37676, GSE79814 and GSE114474. F, G and H Heatmap of the top 40 differentially expressed mRNAs from the GEO microarray GSE37676, GSE79814 and GSE114474 [file 40001_2023_1469_MOESM1_ESM.tif]

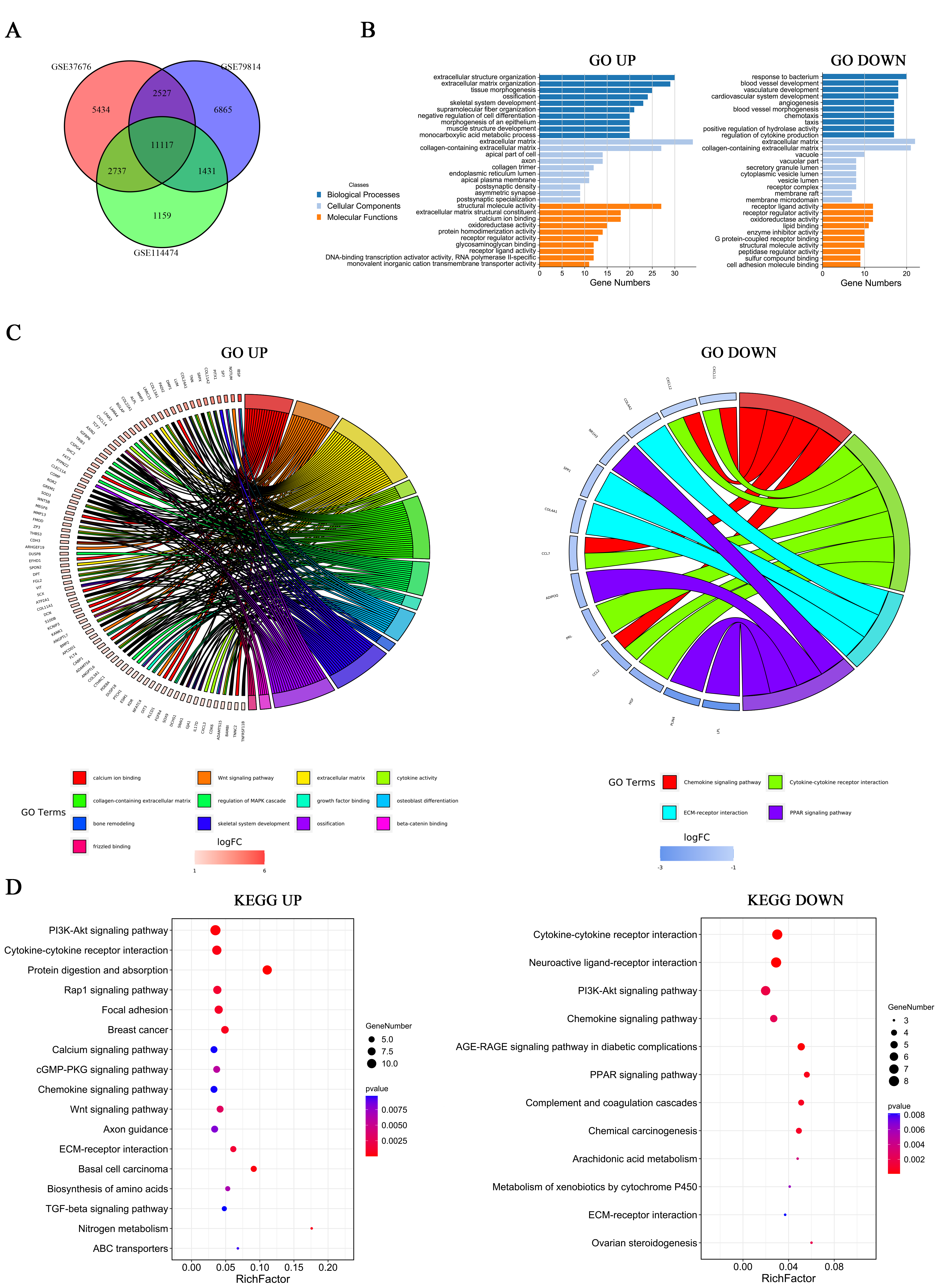

Supplement: Supplementary file 2 — Additional file 2: Figure S2. Functional enrichment analysis of osteogenic All reference citation need to be check manually, abbreviation tagged in normal para tag differentiation-related data sets. A Venn diagram of mRNA in GSE37676, GSE79814 and GSE114474. B Bar plot of GO terms for up/down regulation. GO term name was assigned to y-axis and Gene number was assigned to x-axis. Biological processes (BP), cellular components (CC) and molecular functions (MF) are distinguished by different colors. C GO Chord plot of the relationship between the selected GO terms and their corresponding genes. The left half of the GO chord shows the expression ploidy of the gene (Red is the up-regulated, blue is the down-regulated). The right half represented different GO terms with different colors. D Results of KEGG pathway enrichment analyses for up/down-regulated DEGs. Rich factor = count/pop hits. KEGG Kyoto Encyclopedia of Genes and Genomes, DEGs differently expressed genes, GO gene ontology [file 40001_2023_1469_MOESM2_ESM.tif]
